# Aspartame Causes Developmental Defects and Teratogenicity in Zebra Fish Embryo: Role of Impaired SIRT1/FOXO3a Axis in Neuron Cells

**DOI:** 10.3390/biomedicines12040855

**Published:** 2024-04-12

**Authors:** Athiram Pandaram, Jeyakumari Paul, Wankupar Wankhar, Abhimanyu Thakur, Sakshi Verma, Karthick Vasudevan, Dapkupar Wankhar, Ananth Kumar Kammala, Priyanshu Sharma, Ravindran Jaganathan, Ashok Iyaswamy, Ravindran Rajan

**Affiliations:** 1 Department of Physiology, Dr. ALM PG Institute of Basic Medical Sciences, University of Madras, Chennai 600113, Tamil Nadu, India; 2 Faculty of Paramedical Sciences, Assam down town University, Guwahati 781026, Assam, India; 3 Pritzker School of Molecular Engineering, Ben May Department for Cancer Research, The University of Chicago, Chicago, IL 60637, USA; 4 Department of Pharmacology, Delhi Pharmaceutical Sciences and Research University, New Delhi 110017, India; 5 Department of Pharmacy, Usha Martin University, Ranchi 835103, Jharkhand, India; 6 Department of Biotechnology, REVA University, Bangalore 560064, Karnataka, India; 7 Department of Obstetrics and Gynaecology, The University of Texas Medical Branch, Galveston, TX 77550, USA; 8 Department of Pathology, Massachusetts General Hospital, Harvard Medical School, Boston, MA 02114, USA; 9 Preclinical Department, Faculty of Medicine, Royal College of Medicine Perak, Universiti Kuala Lumpur, Ipoh 30450, Perak, Malaysia; 10 Mr. & Mrs. Ko Chi-Ming Centre for Parkinson’s Disease Research, School of Chinese Medicine, Hong Kong Baptist University, Kowloon Tong, Hong Kong; 11 Department of Biochemistry, Karpagam Academy of Higher Education, Coimbatore 641021, Tamil Nadu, India

**Keywords:** aspartame, zebrafish embryos, teratogenicity, neurodevelopment, SIRT1/FOXO3a axis

## Abstract

Aspartame, a widely used artificial sweetener, is present in many food products and beverages worldwide. It has been linked to potential neurotoxicity and developmental defects. However, its teratogenic effect on embryonic development and the underlying potential mechanisms need to be elucidated. We investigated the concentration- and time-dependent effects of aspartame on zebrafish development and teratogenicity. We focused on the role of sirtuin 1 (SIRT1) and Forkhead-box transcription factor (FOXO), two proteins that play key roles in neurodevelopment. It was found that aspartame exposure reduced the formation of larvae and the development of cartilage in zebrafish. It also delayed post-fertilization development by altering the head length and locomotor behavior of zebrafish. RNA-sequencing-based DEG analysis showed that SIRT1 and FOXO3a are involved in neurodevelopment. In silico and in vitro analyses showed that aspartame could target and reduce the expression of SIRT1 and FOXO3a proteins in neuron cells. Additionally, aspartame triggered the reduction of autophagy flux by inhibiting the nuclear translocation of SIRT1 in neuronal cells. The findings suggest that aspartame can cause developmental defects and teratogenicity in zebrafish embryos and reduce autophagy by impairing the SIRT1/FOXO3a axis in neuron cells.

## 1. Introduction

Aspartame, an artificial sweetener, has become more popular as an alternative to sugar, particularly among those battling obesity, diabetes, metabolic syndrome, and weight loss management [1,2]. However, there is yet to be definitive proof of its safety to back assertions of its use. Aspartame is hydrolyzed in the gastrointestinal (GI) tract into amino acids like aspartic acid, phenylalanine, and methanol [3,4,5]. Our previous studies in rat models suggest that the toxic effects of aspartame are caused by the methanol released during digestion [1,3,5,6].

Methanol is highly toxic and can be absorbed through any route of exposure [3,7]. It quickly spreads throughout the body and accumulates in different tissues because of body fluids’ motion [4,7]. Around 10% of the byproduct is converted into formate, which is either excreted or further metabolized into formaldehyde and other toxic derivatives that can severely damage the brain and liver [4,7,8]. Consumption of aspartame during conception could lead to birth defects due to the high concentrations of phenylalanine in the placenta, which can cause mental retardation and indicate the toxicity of aspartame metabolites [8]. Therefore, there is a need for the study to investigate the role of aspartame in developmental defects and teratogenicity in vivo.

Zebrafish are widely used in developmental studies due to several advantages [9,10]. Firstly, they are relatively easy to maintain in the laboratory and can be bred quickly, producing large numbers of offspring [9,10]. Secondly, they are transparent in their early stages of development, making them suitable for imaging and tracking cell movements [9,10,11]. Thirdly, they have a well-characterized genetic system with many mutants and transgenic lines available. Finally, their genome has been fully sequenced, allowing for powerful molecular genetic studies [9,11]. Moreover, previous reports indicate that the toxicity profiles of both mammalian and zebrafish are strikingly similar [9,11]. This animal model is an effective tool for toxicological research due to its complete organ formation by 120 h post-fertilization (hpf) and its transparent nature, which facilitates easy visualization of phenotypes [11,12,13]. Apparently, the zebrafish can be utilized for examining the potential role of aspartame in developmental defects and teratogenicity in zebrafish embryos. Intriguingly, studies have shown that aspartame can have a negative effect on neurons, including behavioral toxicity, and anti-atherosclerotic activity in zebrafish [14,15]. Specifically, research has demonstrated that aspartame can interfere with the formation of neurons and the release of neurotransmitters, which can lead to neurological disorders [14]. Therefore, it is imperative to employ neuron cells to find a novel mechanism through which aspartame causes aberrant neuronal behavior, leading to developmental defects and teratogenicity.

In this study, we examined the concentration- and time-dependent effects of aspartame on the development of zebrafish, and its possible teratogenicity. Further, sirtuin 1 (SIRT1) and Forkhead-box transcription factor (FOXO) are two proteins that play important roles in neurodevelopment [16,17]. SIRT1 is involved in cell survival and differentiation, while FOXO is involved in apoptosis and cell cycle regulation [17,18]. Defects in SIRT1 and FOXO have been implicated in developmental defects and teratogenicity [16,17]. Studies have shown that SIRT1 and FOXO interact to regulate gene expression and behavior in the developing brain [18]. Abnormal levels of these proteins can lead to cognitive deficits and behavioral impairments, as well as increased susceptibility to teratogenic agents [17,18]. Apparently, understanding the role of these two proteins in neurodevelopment and their involvement in developmental defects and teratogenicity is essential for preventing and treating these conditions [17]. Therefore, we investigated the potential role of aspartame in the impairment of the SIRT1/FOXO3a axis in neuron cells. This would pave the way for developing novel mechanisms underlying the role of aspartame in developmental defects and teratogenicity by utilizing in vitro and in vivo models.

## 2. Materials and Methods

### 2.1. Chemicals and Reagents

Aspartame was obtained from Sigma Aldrich, St. Louis, MO, USA. All major chemicals were obtained from Himedia Laboratories Pvt. Ltd., Mumbai, India. All other chemicals were of analytical grade and obtained from Sisco Research Laboratory, Bombay, India. Anti-Goat IgG HRP conjugated (HAF017) and Goat Anti-Rabbit IgG (111-035-003) antibodies were purchased from Jackson ImmunoResearch Laboratories, PA, USA. Super Signal West Femto (34095) was purchased from Thermo Fisher Scientific, Waltham, MA, USA. Alexa Fluor 488 Goat Anti-Mouse IgG (A11001), Alexa Fluor 594 Goat Anti-Rabbit IgG (A11012), and Alexa Fluor 488 Goat Anti-Rabbit IgG (A11008) were purchased from Thermo Fisher Scientific, Waltham, MA, USA. 10X Tris/Glycine buffer (161–0771), 10X Tris/Glycine/SDS buffer (161–0772), 2X Laemmli sample buffer (161–0737), and Protein Ladder (26616) were purchased from BioRad, CA, USA. Anti- FOXO3a Polyclonal Antibody (PA5-27145), anti-SIRT1 Antibody (MA5-15677), and Anti-Actin Antibody (MA1-744) were purchased from Invitrogen and Thermo Fisher Scientific Waltham, MA, USA. Protease inhibitor (04,693,124 001), Fluorescence mounting media (S3023), 2-Mercaptoethanol (M6250), Phosphate buffered saline (P5368), and Paraformaldehyde (P6148) were purchased from Sigma Aldrich St. Louis, MO, USA.

### 2.2. Animal

Male and female zebrafish (*Danio rerio*) of wild type were purchased from a local fish breeder in Madurai, India. The wild type of zebrafish was preferred over other mutant varieties for accuracy of the study. The fish were maintained in a rectangular glass aquarium at a constant temperature of 25 °C. The fish were fed twice a day with flaked food. The diurnal cycle was maintained as 14 h light and 10 h dark cycles. All the animal experiments were performed according to animal ethical regulations. The work on zebrafish was carried out according to the principles of laboratory care framed by the Committee for the Purpose of Control and Supervision of Experiments on Animals (CPCSEA), Government of India. Prior to the experimentation, ethical approval was obtained from the Institutional Animal Ethical Committee (IAEC) (No: 01/032/2013/June-14).

### 2.3. Breeding of Zebrafish

The zebrafish experiments were conducted in the neurophysiology lab of the Dr. ALM PG IBMS Department of Physiology, University of Madras, Chennai, Tamil Nadu, India; they were approved by the IAEC. To initiate the procedure, the bred fish were fed with red worms for one week, and they were put in an aquarium with plastic mesh at the base to collect the laid eggs and prevent them from getting eaten by the same fish. During mating, the water in the tank was kept at a constant temperature. Three adult males and two females were put in the tank, and eggs were left to fertilize until the next morning. Afterwards, these were transferred to petri dishes containing an embryo buffer medium, where the embryos were cleaned to remove any debris adhered to them. Finally, the fertilized eggs were segregated from the unfertilized eggs and maintained at 25 °C with a light cycling of 14 h light and 10 h dark.

### 2.4. Medium Used for Embryo Development

Hank’s embryo medium is required for the development of zebrafish embryos.

### 2.5. Aspartame Treatment

The tested concentrations of aspartame were weighed separately at 20, 40, 60, 80, and 100 µg/mL. This varying concentration of aspartame was later dissolved in embryonic medium and used to treat the embryos. Adult wild type zebrafish were kept in ad libitum conditions, and embryos were harvested. 60 embryos were taken for the study. They are grouped into 6 groups; each group consists of 10 embryos. Group I—Control, Group II—aspartame treatment (20 µg/mL), Group III—aspartame treatment (40 µg/mL), Group IV—aspartame treatment (60 µg/mL), Group V—aspartame treatment (80 µg/mL), and Group VI—aspartame treatment (100 µg/mL).

### 2.6. Determination of Methanol Level Using HPLC

100 µL of homogenate was deproteinized with an equivalent amount of acetonitrile and centrifuged for 7 min at 4 °C. Afterward, the supernatant (20 µL) was analyzed for methanol and formate using an HPLC refractive index detector system (Shimadzu RID, Kyoto, Japan.) with a Rezex ROA-organic acid column (300 × 7.8 mm Dimension, Phenomenex) and AJO 4490 Phenomenex security guard cartridge. To maintain the temperature at sixty degrees Celsius, a column oven was deployed. The mobile phase was 0.026 N sulfuric acid, with methanol used as an external standard to determine a 92–96% recovery from blood. Linearity of methanol was found to be from five to 500 mg/100 µL, while detector sensitivity and reproducibility were at 5 mg/100 µL and >93%, respectively.

### 2.7. Analysis of Toxicity of Aspartame on Zebrafish Embryo

A series of five ascending concentrations of aspartame (20, 40, 60, 80, and 100 µg/mL) were dissolved in embryonic medium. Following dilution of the stock solution, 1-h post-fertilized eggs were added to 24-well cell culture sterilized plates (10 embryos/well). The embryos were then incubated at 26 °C and monitored daily for four days for any signs of survival, morphological or developmental abnormalities, and toxicity endpoints. Nikon images were captured daily to analyze any morphological anomalies present in the zebrafish embryos. Additionally, any deaths observed at each concentration were recorded and imaged using the Nikon microscope, Tokyo, Japan.

### 2.8. Identification of Developmental Stages of Zebrafish

The developmental stages of zebrafish were categorized, beginning with the zygote period (0–0.45 h post fertilization), followed by cleavage (0.45–2.15 h), blastula (2.15–5.15 h), gastrula (5.15–10 h), segmentation (10–24 h), pharyngula (24–48 h), and concluding with hatching (48–72 h).

### 2.9. Determination of Survival Rate

A standard protocol involving acridine orange staining was used to analyze the survival rate [19]. 28 hpf embryos were dechorionated and incubated in a 10 mg/L acridine orange staining solution for 30 min, then rinsed three times with fish water and mounted with 80% glycerol. Imaging was swiftly conducted via a Nikon microscope (DS-Fi1, Nikon microscope, Tokyo, Japan).

### 2.10. Determination of Somite’s Development

Somite’s development was detected as described previously [20]. Larvae were sampled between 24 and 48 h of development to count the number of somites’ formation or deformation. Somite development was identified, beginning at 10.5 min at a rate of one somite every half an hour.

### 2.11. Analysis of Heart Rate in Zebrafish

The heart rate of zebrafish was measured as described previously [21]. At fertilization, the heartbeat of an embryo was surveyed 24 h post-implantation to measure their heart rate. The embryos were placed beneath a stereomicroscope to observe their heartbeats. The surveys lasted 20 s each, and the resulting data were normalized to beats per minute. This survey was replicated and repeated three times (N = 3).

### 2.12. Measurement of Axial and Head Length of Zebrafish

The head and axial length were measured at various stages, as described previously [22]. An ocular micrometer was used to magnify the measurements and allow for an accurate calculation of the length without magnification. The deformities of the cranio-facial regions and the axial skeleton were then photographed using a Nikon microscope, DS-Fi1, Kyoto, Japan.

### 2.13. Analysis of Yolk Sac Edema

The diameter of the yolk sac is calculated using an ocular micrometer fitted to the microscope, as described previously [23]. To assess the presence of edema in the sac, a Nikon microscope, DS-Fi1, Kyoto, Japan was employed to manually record relevant information. A delay in the development of the embryo is observed when edema is present in the yolk sac.

### 2.14. Analysis of Locomotor Activity

The locomotor activity was analyzed in 96 hpf-stage zebrafish larvae following exposure to concentrations of aspartame ranging from 20 µg to 100 µg, as described previously [24]. Testing was carried out in vitro at 26 ± 0.5 °C with a Nikon microscope (DS-Fi1, Kyoto, Japan). Each treatment was replicated three times in a 6-well culture plate with 10 larvae per replicate. Locomotor responses were monitored manually.

### 2.15. Detection of Cartilage Development in Zebrafish Embryos Using Alcian Blue Staining

The embryonic development of cartilage and bone structures in embryos and complete larvae was observed via Alcian Blue staining, as described previously [25]. Cartilage of bones had started to develop by 48 h after post fertilization and was well demonstrable after four days post fertilization (dpf) upon fixation of the embryos overnight in 4% (p/v) phosphate-buffered paraformaldehyde and maintenance in methanol 100% at −20 °C. Cartilage was then stained using Alcian Blue and left to steep overnight.

### 2.16. Analysis of Protein Levels via Western Blot and Dot Blot

The protein was estimated and equalized from the cell samples and subjected to protein expression using western blot [26]. The loaded protein samples were run on the SDS polyacrylamide gel (SDS-PAGE) and separated as per the target protein requirement. The target proteins were transferred to the PVDF membrane from the gel, and the protein-transfer membrane was incubated in 5% milk for 1–2 h at room temperature with mild shaking. After the blocking process, the target protein in the membrane was incubated with primary antibodies (details listed in the reagent section of methodology) overnight at 4 °C. The next day, the incubated membrane was washed in PBST and subjected to secondary antibody incubation for 1–2 h at room temperature. Then the membrane was washed in PBST and developed on X-ray film using the chemiluminescence (ECL) reagent. The expression levels of the target protein were blotted and quantified using ImageJ software, Version 1.51.

### 2.17. Immunocytochemistry

The treated cells in cover slip were fixed with 4% PFA for 20–30 min and washed with 0.4% PBST for 5–10 min. The target protein primary antibodies (details listed in the reagent section of methodology) were incubated with the treated cells overnight at 4 °C and washed with PBST the next day. The secondary antibody with fluorescence tag was incubated with the washed cells for 1–2 h at room temperature in dark condition. Then the cells in the coverslip were mounted on the slides with fluorescence mounting medium. The cells were pictured under a confocal microscope for target protein expression [27].

### 2.18. In Vitro Cell Culture and Cell Treatment

Mouse neuroblastoma cells (N2a) and N2a cells overexpressing tandem fluorescent-tagged LC3 (tfLC3) were used in the neuronal and autophagy flux-elated experiments [28]. The transfected cells were treated with aspartame (100 µM), CQ (20 µM), and Torin1 (250 nM) in separate cover glasses for 24 h, then the cells were fixed and embedded in the slides for confocal microscopy imaging of GFP and RFP LC3. The colocalization was assessed using ImageJ analysis.

Additionally, for the Sirt1 nuclear translocation experiment, the N2a cells were treated with aspartame (100 µM) and Resveratrol (0.1 µM) for 24 h, and then the cells were fixed and immunocytochemistry was carried out for confocal microscopy imaging of nuclear translocation of Sirt1. The colocalization was assessed using ImageJ analysis.

The N2a cells were maintained in DMEM media supplemented with 5–10% fetal bovine serum (FBS) and 50–100 units of PSN mixture for most of the treatment experiments [29]. After 24 h of growth, the N2a cells were treated with or without aspartame at different concentrations (0, 60, 80, and 100 µM) for 24 h, then the cells were scrapped in the 1X RIPA buffer and analyzed for protein after a quick sonication in the cell lysate-containing tube. The protein was normalized and detected using western blot and dot blot for the sirt1 and foxo3a proteins, and its expression levels were analyzed using ImageJ analysis.

### 2.19. Transmission Electron Microscopy (TEM)

The TEM analysis of cells was conducted as described previously [30]. The treated cells were fixed in 2.5% glutaraldehyde in 0.1 M sodium cacodylate buffer for 1 h at room temperature. The fixed cells were rinsed in 0.1 M sucrose in cacodylate buffer, post-fixed with 1% osmium tetroxide for dehydration, and mounted for sectioning using an ultramicrotome. The sliced cells were fixed in carbon-coated electron microscopy grids and viewed under a Philips CM100 transmission electron microscope for taking pictures and quantitative analysis. Each treated (aspartame (100 µM) and Torin1 (250 nM)) cell was photographed for 5 to 10 images at different regions of N2a cells. The photographed micrograph was analyzed for the mature autolysosome using ImageJ analysis and manually to conform to the selection of mature autolysosome. Three to five experimental repeats were used to confirm the results, and statistical analysis was carried out using GraphPad Prism software, Version 8.3.0(538). One-way ANOVA was performed, followed by post-hoc comparison of the means using Bonferroni’s or Dunnett’s T3 methods.

### 2.20. Dataset

In this study, we conducted a thorough analysis to explore the contribution of Foxk proteins to zebrafish embryonic development. Our approach involved using a publicly available dataset with the GEO accession number PRJEB41393 [31]. To produce the count data essential for differential gene expression analysis, we obtained high-throughput sequencing reads, which were evaluated using FastQC to ensure quality.

### 2.21. Differential Gene Expression Analysis

To assess the differential gene expression in zebrafish embryonic development, we aligned the obtained reads to the reference genome of zebrafish (*Danio rerio*) and quantified them using feature counts [32,33], resulting in raw counts for each gene. These counts were then supplied as input for the DESeq2 package 3.18 within the Bioconductor project implemented in R, taking the shape of a matrix of integer values representing unnormalized or estimated counts of sequencing reads for each gene. Employing empirical Bayes techniques to estimate log fold change (logFC) priors and dispersion enabled the identification of genes with significant differential expression. A logFC cutoff of +2.5 and −2.5 distinguished genes as upregulated and downregulated, respectively. This approach allowed for the visualization, analysis, and comparison of high-dimensional count data, thus identifying robust and biologically relevant differentially expressed genes associated with the knockout of Foxk proteins in zebrafish embryonic development.

### 2.22. Analysis of Protein-Protein Interaction via STRING Database

To explore the protein-protein interactions and gain insights into the functional implications of the differentially expressed genes, STRING database was utilized as described previously [34,35]. This database is a valuable resource containing known and predicted protein-protein interactions, integrating curated data from sources such as experiments, data mining, literature, and gene co-expression studies. STRING assigned confidence scores to the interactions, reflecting the strength of evidence supporting them, which were categorized into four levels: highest, high, medium, and low. Leveraging this information, we analyzed and interpreted the potential functional partners and networks associated with our differentially expressed genes. To categorize the functional partners of these interactions, probabilistic confidence scores were used. The confidence scores lie between 0 and 1, and the more evidence there is, the higher the confidence scores. String classifies the interactions into four categories based on the confidence scores: highest (above 0.90), high (0.7–0.89), medium (0.4–0.69), and low (0.15–0.39). In the database channel, manual curation is performed on interaction records gathered from KEGG, Reactome, BioCyc, and Gene Ontology, as well as legacy datasets from PID and BioCarta. In STRING, associations are only stored within a protein complex or between members of the direct pathway.

### 2.23. Enrichment Analysis Using ClueGO

To investigate the functional enrichment and biological processes linked to our gene set, we employed the ClueGO plugin [36] within the Cytoscape software, Version 3.8.2. ClueGO enabled us to visualize functionally grouped networks based on kappa statistics, which measure the similarity between terms associated with genes. Utilizing precompiled annotation files for gene ontology (GO), Kyoto Encyclopedia of Genes and Genomes (KEGG), and BioCarta, we were able to perform a comprehensive analysis across a wide range of organisms. ClueGO utilized the hypergeometric distribution to conduct enrichment/depletion tests of the terms and groups associated with our gene set. Hence, we applied standard correction methods such as Bonferroni, Bonferroni step-down, and Benjamini-Hochberg in order to account for multiple testing [37].

### 2.24. Single Cell Transcriptomic Analysis

The single-cell RNA-seq data of developing zebrafish hindbrain (NCBI’s GEO accession: GSE141428) [38] was analyzed to generate UMAP and violin plots showing cell-type-specific and developmental stage-specific expression of SIRT1 and FOXO3A as described previously [39].

### 2.25. Molecular Docking Analysis

The molecular docking approach was conducted to assess the binding affinity and key interactions between the target protein and compounds. The 3D structures of SIRT1 were modeled using Modeler, and quality was verified through a Ramachandran plot. The aspartame compound information was retrieved from PubChem and converted to a 3D structure through Open Babel. AutoDockTools (version 1.5.6) was utilized to add hydrogen atoms and compute the Kollman charges [40]. The binding site information was retrieved from literature evidence and the LPC CSU Server (https://oca.weizmann.ac.il/oca-bin/lpccsu) accessed on 13 May 2023 [41]. The pdb format of both the target protein and compounds was then converted to pdbqt format. AutoDock Vina was employed to execute the molecular docking analysis between the target protein and compound [42]. To guarantee the consistency of the molecular docking result and determine the best binding pose, the process was repeated. The key intermolecular interactions and binding modes were depicted through BIOVIA Discovery Studio v.2021 and Chimera v.1.16 [43].

### 2.26. Statistical Analysis

Data from the somites’ development, heart rate, axial length, head length, yolk sac edema, and locomotor activity are analyzed by one-way analysis of variance, and when there is a significant *t*-test ratio, it is followed by Tukey’s multiple comparison by fixing significance at *p* < 0.05 level. All analyses were performed using GraphPad Prism software, Version 8.3.0(538). For in vitro experiments, a one-way ANOVA was performed, followed by post-hoc comparison of the means using Bonferroni’s or Dunnett’s T3 methods. Data were presented as the mean ± S.E.M.

## 3. Results

### 3.1. Aspartame Exposure Reduces the Formation of Larvae and the Development of Cartilage in Zebrafish

**Figure 1:**
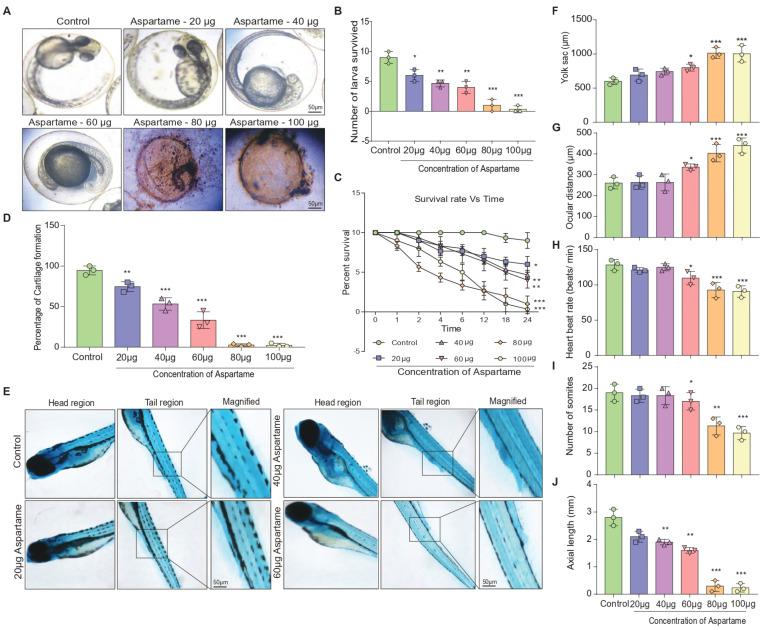
Aspartame affects the formation of larvae and the embryonic developmental characteristics of zebrafish. (**A**) The representative images illustrate the number of survived larvae during the 24 h of aspartame treatment at varying concentrations. (**B**,**C**) Quantification of (**B**) number of survived eggs and (**C**) percentage of survival rate during the 24 h timepoint. (**D**,**E**) Quantification of the Zebra fish larva cartilage formation (N = 10) on exposure to varying concentration of aspartame using Alcian Blue staining, as shown in the representative Figure (**E**). (**F**–**J**) Quantification of the Zebra fish’s (**F**) yolk sac diameter (N = 10), (**G**) ocular distance, (**H**) heart rate for 24 h, (**I**) somite formation for 48 h, and (**J**) axial length for 48 h on exposure to varying concentrations of aspartame compared with the control group. The data were presented as mean ± SEM (N = 10). Each experiment has been repeated with three individual trials. Comparison and analysis were done using ANOVA with the statistical significance fixed at * *p* ˂ 0.05, ** *p* ˂ 0.01, and *** *p* ˂ 0.001.

Using a zebrafish model, the present study sought to examine the effect of aspartame, which has become increasingly common in recent years, on the formation of larvae and the development of cartilage. Aspartame has been approved for use by the FDA in certain products and has raised concern over its potential effects on child development [44]. Figure 1A shows representative brightfield images of typical larvae of zebrafish after the treatment of aspartame at different concentrations (20, 40, 60, 80, and 100 μg) for 24 h. Results showed that the survival of larvae decreased with increased concentrations of aspartame, as shown by a quantitative bar graph as well as a time-dependent survival curve (Figure 1B,C). Furthermore, the formation of zebrafish larvae cartilage in the head and tail regions was monitored using Alcian Blue staining after treatment with aspartame at different concentrations. Results showed that the percentage of cartilage formation was reduced as the concentration of aspartame increased (Figure 1D,E). Other analyses revealed alterations in the size of the yolk sac, ocular distance, heartbeat rate, number of somites’ formations, and axial length (Figure 1F–J). Moreover, the aspartame metabolite methanol was found to be released in embryo homogenates, as shown in HPLC analysis (Supplementary Table S1), which is highly toxic for the development of embryos. Taken together, these results suggest that aspartame is toxic, and an increase in its concentration may lead to the death of larvae. Despite the FDA’s recommended daily allowances of 40 mg/kg bodyweight in Europe and 50 mg/kg bodyweight in the United States [44], further caution should be taken when using aspartame in food and other products.

### 3.2. Aspartame Delays Post-Fertilization Development by Altering the Head Length and Locomotor Behavior of Zebrafish

**Figure 2:**
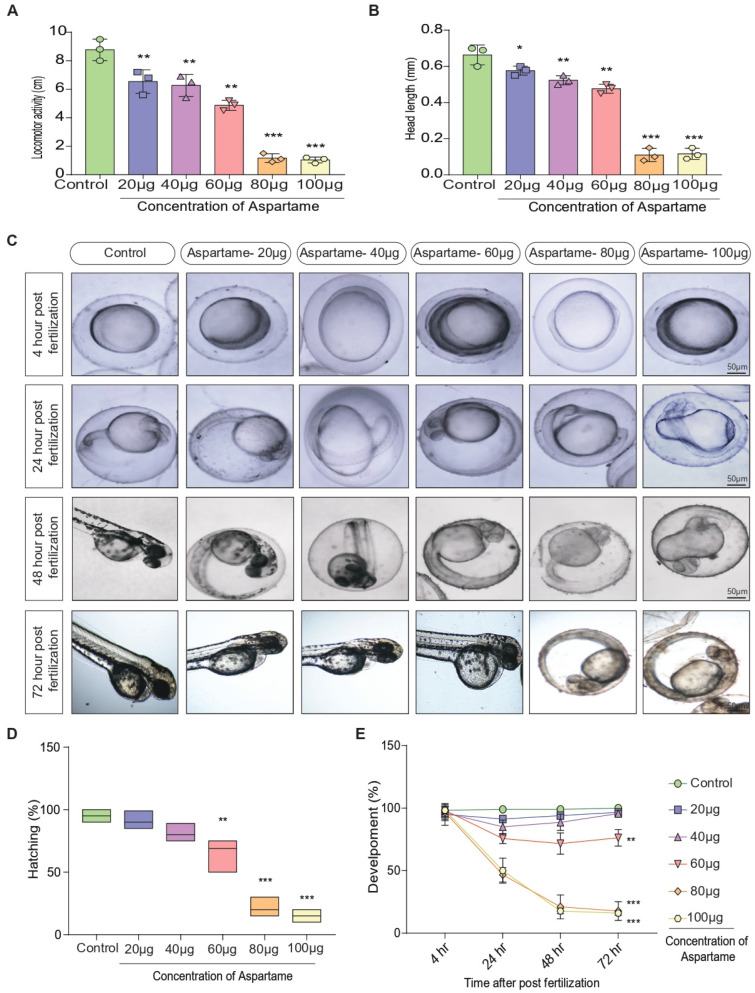
Aspartame affects head length, locomotion, hatching, and delays post-fertilization embryonic development in zebrafish. Representative (**A**,**B**) quantitative bar graph showing the effect of different concentrations of aspartame on the locomotor activity (swimming) in zebrafish embryos (96 h post fertilization) and the head length of the embryo. (**C**) Representative images illustrate the time-dependent decrease in the development of the zebrafish embryo, and (**D**) the quantitative bar graphs show the effect of different concentrations of aspartame on the hatching efficiency of the zebrafish embryo, causing (**E**) the time-dependent (4, 24, 48, 72 h post fertilization) decrease in development. The data were presented as mean ± SEM (N = 10). Each experiment has been repeated with three individual trials. Comparison and analysis were done using ANOVA with the statistical significance fixed at * *p* ˂ 0.05, ** *p* ˂ 0.01, and *** *p* ˂ 0.001.

Multiple studies have assessed the effect of delayed post-fertilization development on zebrafish behavior and development [45]. In one such investigation, zebrafish embryos were exposed to variable temperatures, and those exposed to lower temperatures evidenced a reduction in head length and delayed development [46]. Such a delay in development had consequent effects on zebrafish behavior, including decreased mobility and slower swimming speeds. Subsequently, another study reported that delayed development influenced the neuromuscular junctions of zebrafish with regard to muscle function and locomotive activities [47]. Apparently, it is pertinent to examine the potential effect of aspartame on the post-fertilization delay. Interestingly, the concentration of aspartame (Figure 2A,B) was found to correlate with a decrease in locomotor activity, head length, and hatching of zebrafish embryos (Figure 2C–E). Such a finding implies that aspartame may impede the embryonic development of zebrafish by delaying post-fertilization, as indicated by the successive decline in head length, locomotion, and hatching. In totality, these investigations underline the importance of considering the effects of delayed post-fertilization development on zebrafish behavior and development.

### 3.3. RNA-Sequencing-Based DEG Analysis Shows SIRT1 and FOXO3a Are Involved in the Neurodevelopment

**Figure 3:**
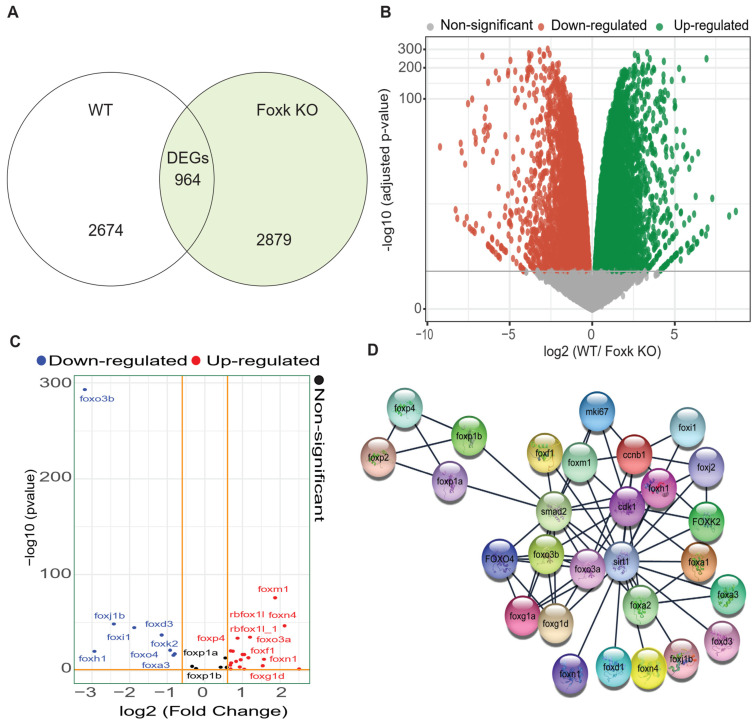
RNA-seq-based DEG analysis showed SIRT1/FOXO3a as crucial signaling in the neurodevelopment of zebrafish. (**A**) Venn diagram demonstrates the differentially regulated genes in the wild type (WT) and Foxk knockout (KO) zebrafish models for the development of zebrafish embryos. (**B**) The volcano plot shows the down-regulated and up-regulated genes among the WT and Foxk KO zebrafish models for the development of zebrafish embryos and their quantification. (**C**) A simplified volcano plot illustrating the DEGs of FoxO family genes and their fold change comparison involved in the development of zebrafish embryos. (**D**) The protein-protein interaction network analysis showed the interaction of the SIRT1 and FoxO family proteins involved in the development of zebrafish embryos when compared with the WT and Foxk KO zebrafish models.

Previously, it has been found that Foxk, a transcriptional regulator, plays a critical role in the development and function of the nervous system [31]. They control the expression of multiple genes, including those involved in neuronal migration, axon guidance, axon branching, and synaptic transmission [31]. In recent years, Foxk proteins have been found to be involved in genetic diseases related to neurodevelopment, such as autism spectrum disorder and epilepsy. By regulating gene expression, Foxk proteins can help ensure that the developing brain has the appropriate number and types of neurons and that these neurons have the correct connections and functions [31]. Notably, the depletion of Foxk transcription factors has been found to cause genome-wide transcriptional misregulation and developmental arrest in zebrafish embryos [31]. Therefore, we examined the microarray dataset (Project: PRJEB41393, Secondary Study Accession: ERP125162) from the public database, the European Nucleotide Archive (ENA). Importantly, 964 differentially expressed genes (DEGs) were found between WT and Foxk KO, as evident from the Venn diagram and the volcano plot (Figure 3A,B). Supplementary Figure S1 shows the top DEGs between the WT and Foxk KO groups of zebrafish embryos, and Supplementary Figure S2 shows the ClueGo visualization of a gene ontology interaction network. Among the top up-regulated genes, FOXO3a was also found to be up-regulated (Figure 3C, Supplementary Figure S3), which was also supported by the STRING analysis-based protein-protein interaction. Intriguingly, FOXO3A was also found to be closely interacting with SIRT1 (Figure 3D). Analysis of single-cell RNA-seq data of developing zebrafish hindbrain (NCBI’s GEO accession: GSE141428) [38] also revealed the different cell types and expression of SIRT1 and FOXO3A in different cell types and based on the developmental stages, namely 16, 24, and 44 hpf (Supplementary Figure S4A–H). SIRT1 and FOXO3a are key proteins that regulate neurodevelopment. SIRT1 is a protein deacetylase that can regulate gene expression, while FOXO3a is a transcription factor that can control neuronal differentiation and growth. Together, these proteins play a role in the development of neurons by promoting cell survival, reducing inflammation, and modulating synaptic plasticity. Additionally, FOXO3a is involved in the regulation of axon outgrowth and dendritic spine formation, which are essential processes for establishing neuronal circuits. Thus, SIRT1 and FOXO3a are important regulators of neurodevelopment [48,49,50]. Research has demonstrated that the class III histone deacetylase SIRT1 can reduce the apoptotic activity of FOXO3 in CGNs by deacetylating FOXO3 at Lys242 and Lys245, a process that is also shown to occur on FOXO1 at the same sites [49,50,51,52]. SIRT1 is known to be triggered by reactive oxygen species (ROS) and stressors like DNA damage [50,53]. It will be intriguing to delve further into the relationship between these changes and how they work together to control the function of FOXO and SIRT1 and neuronal survival [53,54].

### 3.4. Aspartame Could Target and Reduce the Expression of SIRT1 and FOXO3a Proteins in Neuron Cells: In Silico and In Vitro Evidence

**Figure 4:**
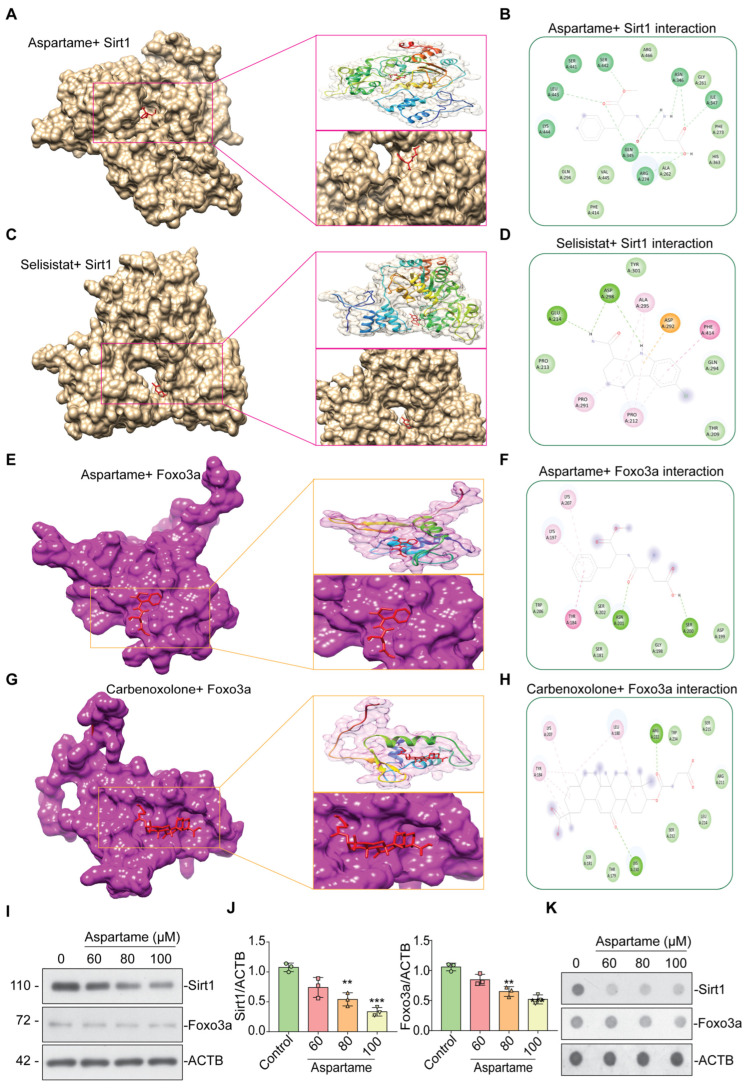
Aspartame inhibits SIRT1 and FOXO3a in neuronal cells. (**A**) Molecular docking picture reveals aspartame binds to the ligand binding domain of Srit1 and inhibits its activity. The magnified image of the molecular docking picture reveals aspartame binds to the ligand binding domain of SIRT1. (**B**) The residues involved in the intact binding of aspartame to the ligand binding domain of SIRT1 reveal its inhibitory effect. (**C**) Molecular docking picture reveals Selisistat (positive control) binds to the ligand binding domain of Srit1 and inhibits its activity. (**D**) The residues involved in the intact binding of Selisistat (positive control) to the ligand binding domain of SIRT1 reveal its inhibitory effect. (**E**) Molecular docking picture reveals aspartame binds to the ligand binding domain of FOXO3a and inhibits its activity. The magnified image of the molecular docking picture reveals aspartame binds to the ligand binding domain of FOXO3a. (**F**) The residues involved in the intact binding of aspartame to the ligand binding domain of FOXO3a reveal its inhibitory effect. (**G**) Molecular docking picture reveals Carbenoxolone (positive control) binds to the ligand binding domain of FOXO3a and inhibits its activity. (**H**) The residues involved in the intact binding of Carbenoxolone (positive control) to the ligand binding domain of FOXO3a reveal its inhibitory effect. (**I**) Aspartame treatment at different concentrations reduced the protein concentration of SIRT1 and FOXO3a in neuronal cells using western blot. (**J**) The quantification of the protein concentration of SIRT1 and FOXO3a in neuronal cells. (**K**) The dot blot assay confirmed that aspartame treatment at different concentrations reduced the protein concentration of SIRT1 and FOXO3a in neuronal cells. Data in F was presented as the mean ± SEM (N = 3). Comparison and analysis were done using ANOVA with the statistical significance fixed at ** *p* ˂ 0.01, and *** *p* ˂ 0.001.

Our bioinformatic analysis (Figure 3A–D) as well as previous reports [55,56,57,58] suggest the involvement of SIRT1 and FOXO3a in neuronal health. During neuronal development, SIRT1 has been found to promote axonal elongation, neurite outgrowth, and dendritic branching. SIRT1 also plays a role in memory formation by modulating synaptic plasticity. SIRT1 plays protective roles in several neurodegenerative diseases, including Alzheimer’s, Parkinson’s, and motor neuron diseases, which may relate to its functions in metabolism, stress resistance, and genomic stability [56,58,59]. FOXO3a deacetylation was another SIRT1 target implicated in promoting cell survival in Huntington’s disease models [56]. Apparently, it is pertinent to examine the potential effect of Apartame on SIRT1 and FOXO3a, which are involved in neurodevelopment. Therefore, we performed in silico molecular docking of aspartame with SIRT1 (Figure 4A,B) and compared it with positive control molecules (Selisistat for SIRT1) (Figure 4C,D). Similarly, molecular docking of aspartame with FOXO3a (Figure 4E,F) and comparing it with positive control molecules (Carbenoxolone for FOXO3a) (Figure 4G,H). Notably, as compared with Selisistat, aspartame was found to be firmly binding with SIRT1 via various residues, including ARG-274, GLN-345, ASN-346, ILE-347, SER-442, and LEU-443 (Table 1). In addition, aspartame was also found to bind with FOXO3a via different residues, including SER-200 and ASN-201, although the binding energy was found to be less compared with that for Carbenoxolone binding to FOXO3a (Table 1). To further validate the in silico results, we performed western blot and dot blot, which showed that aspartame could reduce the protein level of SIRT1 as well as FOXO3a in a concentration-dependent manner (Figure 4I–K). Evidently, aspartame can potentially interact with SIRT1 and FOXO3a and reduce their expression, as evident from in silico molecular docking analysis and experimental validation with immune-blotting analysis.

### 3.5. Aspartame Triggered the Autophagy Flux Reduction by Inhibiting Nuclear Translocation of SIRT1 in Neuronal Cells

**Figure 5:**
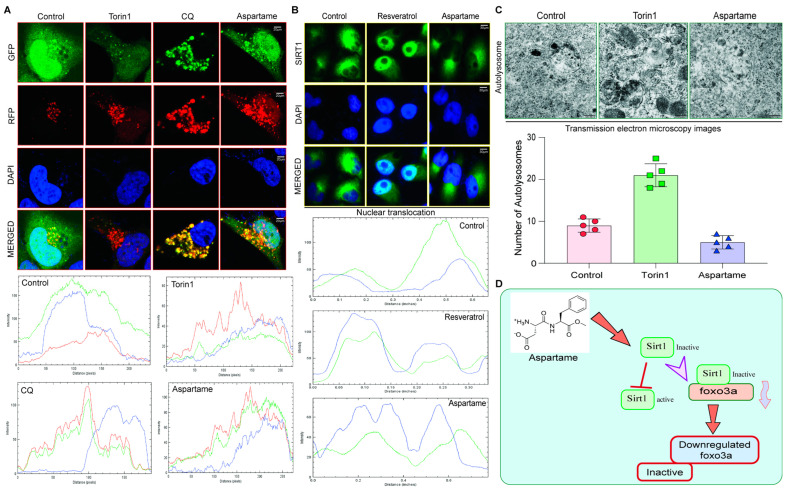
Aspartame inhibits the nuclear translocation of SIRT1 to reduce the autophagy flux in neuronal cells. (**A**) Representative immunocytochemistry staining revealed that the treatment of aspartame in N2a neuronal cells (transfected with stably expressing tf-LC3 plasmids for 48 h) inhibited autophagy flux when compared with Torin1 (positive control) and CQ (negative control). GFP is the generated autophagosomes, and RFP is the autolysosomes, which are the fused autophagosomes and lysosomes. (**B**) The cell staining experiment revealed that aspartame inhibited the activity of SIRT1 and its nuclear translocation in N2a neuronal cells when compared with the positive control, resveratrol. (**C**) Transmission electron microscopy revealed that aspartame treatment reduced autolysosome formation and inhibited autophagy in N2a neuronal cells. (**D**) The schematic diagram demonstrates that aspartame treatment at varying concentrations caused developmental defects and teratogenicity in the zebrafish embryo via the SIRT1/FOXO3a axis.

As we found that aspartame could bind to SIRT1 and FOXO3a (Figure 4A–K), we intended to examine its potential effect on the autophagy flux in neuronal cells because FoxOs in conjunction with SIRT1 and mTOR can modulate autophagy activation and offer beneficial outcomes [16,55]. Supplementary Figure S5 shows the in silico pathway analysis, which also suggests the involvement of the SIRT1/FoxO family in the development of zebrafish embryos. Moreover, FoxO function is essential for the maintenance of autophagic flux and neuronal morphogenesis in adult neurogenesis [60]. Autophagy flux is a process in which dysfunctional or superfluous proteins are degraded by special lysosomes found within the cell [61]. By maintaining autophagy flux, SIRT1 and FOXO3a play important roles in promoting neurodevelopment. They ensure the necessary proteins and signals are available while also preventing the accumulation of potentially toxic substances [60]. Therefore, we examined if aspartame could affect SIRT1-dependent autophagy flux in neuronal cells. Notably, it was found that aspartame blocked the fusion of generated autophagosomes and lysosomes, which hampered the autophagy flux process, causing the toxic accumulation of dead organelles and proteins. Torin 1 (positive control) enhanced red-only puncta of LC3-II protein (autolysosomes) when compared with aspartame, indicating that Torin1 promotes the formation of autophagosomes and fusion with lysosomes, but the fusion was blocked when the N2a cells were treated with chloroquine (CQ) and aspartame (Figure 5A). At the same time, the nuclear expression of SIRT1 was blocked by aspartame, contrary to the effect of Resveratrol, which was used as a positive control group, confirming the validity of the experiment (Figure 5B). This was also accompanied by a reduction in the number of autolysosomes formed (Figure 5C), indicating the inhibition of autophagy in neuronal cells. Evidently, the use of aspartame may potentially affect autophagy-dependent neurodevelopment via SIRT1/FOXO3a signaling (Figure 5D).

## 4. Discussion

Aspartame is one of the most popular low-calorie artificial sweeteners and is commonly used in a variety of foods and beverages. A recent study found that exposure to aspartame reduced the formation of larvae and the development of cartilage in zebrafish [15,62]. Research has revealed that aspartame causes a decrease in the maturation of larvae due to impaired neurodevelopment. There was also an effect on the development of cartilage, leading to poorer skeletal development [15,62]. These findings suggest that the regular consumption of aspartame may be detrimental to the development of animals, such as zebrafish. Intriguingly, our results showed that increased concentrations of aspartame had adverse effects on zebrafish larvae. Quantitative bar graphs and a time-dependent survival curve (Figure 1A–C) showed decreased survival. Staining data showed reduced formation of cartilage (Figure 1D,E). Yolk sac size, ocular distance, heartbeat rate, number of somites’ formation, and axial length were all altered (Figure 1F–J). These suggest that aspartame is toxic, and an increase in concentration can lead to death.

Recent studies have found that it can have a significant effect on the post-fertilization development and behavior of zebrafish [7,14,15]. The study showed that embryonic zebrafish exposed to aspartame experienced a decrease in head length and locomotor behavior as compared with those not exposed. This suggests that aspartame can delay development by altering developmental processes [7,14]. The long-term effects of this delay are unknown; however, this research is important for understanding the environmental influences on the development and behavior of zebrafish. Notably, our data shows that greater aspartame exposure led to poorer locomotion, a decrease in head length, and delayed hatching in zebrafish embryos (Figure 2A–E). This suggests aspartame impairs embryonic development by hindering post-fertilization. These findings affirm the importance of investigating delayed post-fertilization effects on zebrafish behavior and growth.

Bioinformatics analysis of RNA-seq data is important for exploring the involvement of target genes in various biological phenomena. Through bioinformatics approaches, such as gene expression profiling, we can identify target genes that are likely to play a role in crucial aspects of development and unravel complex biological processes. RNA-seq data can also be used to understand the mechanisms of gene regulation and to discover new genetic interactions between different genes [31,63]. Our analysis of RNA seq data (available publicly) revealed that two key proteins involved in neuronal development, SIRT1 and FOXO3a, interact closely [31]. Venn and volcano plots of DEGs between WT and Foxk KO show 964 DEGs, and FOXO3a is markedly upregulated [31]. STRING analysis confirms this. SIRT1 deacetylates RNA, and FOXO3a regulates neuron growth/differentiation (Figure 3A–D).

SIRT1 plays important roles in neuronal development, promoting axonal elongation, neurite outgrowth, and dendritic branching [56,58]. In addition, it is essential for memory formation and plays a protective role in several neurodegenerative diseases [55,56]. FOXO3a deacetylation is an important SIRT1 target for cell survival in Huntington’s disease models [16,50,58]. This inspired us to investigate the effect of aspartame on SIRT1 and FOXO3a through in silico molecular docking, western blot, and dot blot. With Selisistat for SIRT1 and Carbenoxolone for FOXO3a as positive control molecules, aspartame showed strong binding ability towards SIRT1. Aspartame could also bind to FOXO3a, although with relatively less affinity compared with Carbenoxolone (Figure 4A–H, Table 1). Additionally, the immune-blotting results showed that aspartame can reduce the protein levels of both SIRT1 and FOXO3a in a concentration-dependent manner. In conclusion, aspartame has the ability to interact with SIRT1 and FOXO3a and reduce their protein levels (Figure 4I–K).

Further, we sought to determine if aspartame, which can bind to SIRT1 and FOXO3a, decreases autophagy flux in neuronal cells. Autophagy flux is an important process that ensures the necessary proteins and signals are available and prevents the accumulation of toxins [29,60,64]. It was found that aspartame blocked the fusion of autophagosomes and lysosomes, which interfered in the autophagy flux process, causing the toxic accumulation of dead organelles and proteins. Although aspartame activated the formation of autophagosomes, nuclear expression of SIRT1 was blocked and the formation of autolysosomes was reduced (Figure 5A–D). Conclusively, these results demonstrate that aspartame may inhibit autophagy by affecting SIRT1/FOXO3a signaling.

## 5. Conclusions

This study unfolded the impact of aspartame on zebrafish development and teratogenicity, focusing on the concentration- and time-dependent effects. By examining the involvement of SIRT1 and FOXO proteins in neurodevelopment, we observed that aspartame exposure hindered larval formation, cartilage development, and post-fertilization progress in zebrafish. Through RNA-sequencing analysis, we identified the role of SIRT1 and FOXO3a in neurodevelopment, with in silico and in vitro assessments revealing aspartame’s ability to diminish their expression in neuronal cells. Notably, aspartame disrupted autophagy flux by impeding SIRT1 nuclear translocation. These findings underscore the potential of aspartame to induce developmental abnormalities and teratogenic effects in zebrafish embryos while compromising autophagy through the SIRT1/FOXO3a axis in neuron cells.

## Figures and Tables

**Table 1:** Binding energies and the residues involved in the interaction between different ligands and their respective receptors.

Ligand	Binding Energy (Kcal/mol)	Binding Residues
SIRT1 vs. Aspartame	−7.4	ARG-274, GLN-345, ASN-346, ILE-347, SER-442, LEU-443
SIRT1 vs. Selisistat	−7.0	GLU-214, ASP-298
FOXO3a vs. Aspartame	−5.4	SER-200, ASN-201
FOXO3a vs. Carbenoxolone	−7.2	ARG-222, LYS-230

## Data Availability

All the data relevant to this manuscript are presented here, and additional information or raw data can be obtained upon reasonable request from the corresponding authors.

## Supplementary Materials

The following supporting information can be downloaded at: https://www.mdpi.com/article/10.3390/biomedicines12040855/s1, Figure S1: Heat map analysis showing the top differentially expressed genes in the WT vs. Foxk KO zebrafish embryo.; Figure S2: ClueGo visualization of a gene ontology interaction network. Figure S3: Heat map analysis showing the top differentially expressed Foxo family-associated genes in comparison to wild type and Foxk KO. Figure S4: Single-cell transcriptomic analysis of the zebrafish hindbrain. Figure S5: The KEGG pathway analysis in STRING for protein interaction illustrates the FOXO signaling pathway. Table S1: Determination of methanol level at different time point intervals in the embryo homogenates subject to aspartame treatment at different concentrations.
