# Supplementary material for: Aspartame Causes Developmental Defects and Teratogenicity in Zebra Fish Embryo: Role of Impaired SIRT1/FOXO3a Axis in Neuron Cells
Source: Biomedicines. 2024 Apr 12;12(4):855. doi: 10.3390/biomedicines12040855 (PMC11048232; doi:10.3390/biomedicines12040855)
Supplement: Supplementary file 1 [file biomedicines-12-00855-s001.zip › biomedicines-2928830-supplementary.pdf]

Supplementary Data

Aspartame Causes Developmental Defects and Teratogenicity in Zebra Fish Embryo: Role of Impaired SIRT1/ FOXO3a Axis in Neuron Cells

Athiram Pandaram [1,†], Jeyakumari Paul [1,†], Wankupar Wankhar [2,†], Abhimanyu Thakur [3,4,†,*], Sakshi Verma [5], Karthick Vasudevan [6], Dapkupar Wankhar [2], Ananth Kumar Kammala [7], Priyanshu Sharma [8], Ravindran Jaganathan [9], Ashok Iyaswamy [10,11,*] and Ravindran Rajan [1,*]

[1] Department of Physiology, Dr. ALM PG Institute of Basic Medical Sciences, University of Madras, Chennai 600113, Tamil Nadu, India
[2] Faculty of Paramedical Sciences, Assam down town University, Guwahati 781026, Assam, India
[3] Pritzker School of Molecular Engineering, Ben May Department for Cancer Research, The University of Chicago, IL 60637, USA
[4] Department of Pharmacology, Delhi Pharmaceutical Sciences and Research University, New Delhi 110017, India
[5] Department of Pharmacy, Usha Martin University, Ranchi 835103, Jharkhand, India
[6] Department of Biotechnology, REVA University, Bangalore 560064, Karnataka, India
[7] Department of Obstetrics and Gynaecology, The University of Texas Medical Branch, Galveston, TX 77550, USA
[8] Department of Pathology, Massachusetts General Hospital, Harvard Medical School, Boston, MA 02114, USA
[9] Preclinical Department, Faculty of Medicine, Royal College of Medicine Perak, Universiti Kuala Lumpur, Ipoh 30450, Perak, Malaysia
[10] Mr. & Mrs. Ko Chi-Ming Centre for Parkinson's Disease Research, School of Chinese Medicine, Hong Kong Baptist University, Kowloon Tong, Hong Kong
[11] Department of Biochemistry, Karpagam Academy of Higher Education, Coimbatore 641021, Tamil Nadu, India
* Correspondence: abithakur1211@gmail.com (A.T.); iashok@hkbu.edu.hk (A.I.); ravindran89@gmail.com (R.R.)
† These authors contributed equally to this work.

Supplementary Table S1. Determination of methanol level at different time point intervals in the embryo homogenates subject to aspartame treatment at different concentrations.

Data are expressed as Mean ± SEM and statistical significance was fixed at *p<0.0; significant compared to control and aspartame concentrations (N=10). Each experiment has been repeated with three individual trials.

Parameters	Control	Aspartame 20 µg/ml	Aspartame 40 µg/ml	Aspartame 60 µg/ml	Aspartame 80 µg/ml	Aspartame 100 µg/ml
	Concentration of methanol in embryo homogenates (µM)					
**0 hours**	0.004±0.01	0.98±0.03	0.89±0.01	0.97±0.02	1.84±0.04*	1.97±0.03*
**24 hours**	0.001±0.02	1.18±0.02	1.89±0.01	2.69±0.03	5.60±0.06*	7.75±0.06*
**48 hours**	--	1.38±0.04	2.56±0.03	3.89±0.02	6.42±0.05*	8.00±0.05*
**72 hours**	0.001±0.01	1.12±0.01	2.32±0.02	3.45±0.02	5.69±0.03*	7.99±0.07*
**96 hours**	0.002±0.01	0.96±0.03	1.69±0.01	3.12±0.01	6±0.01*	7.19±0.09*

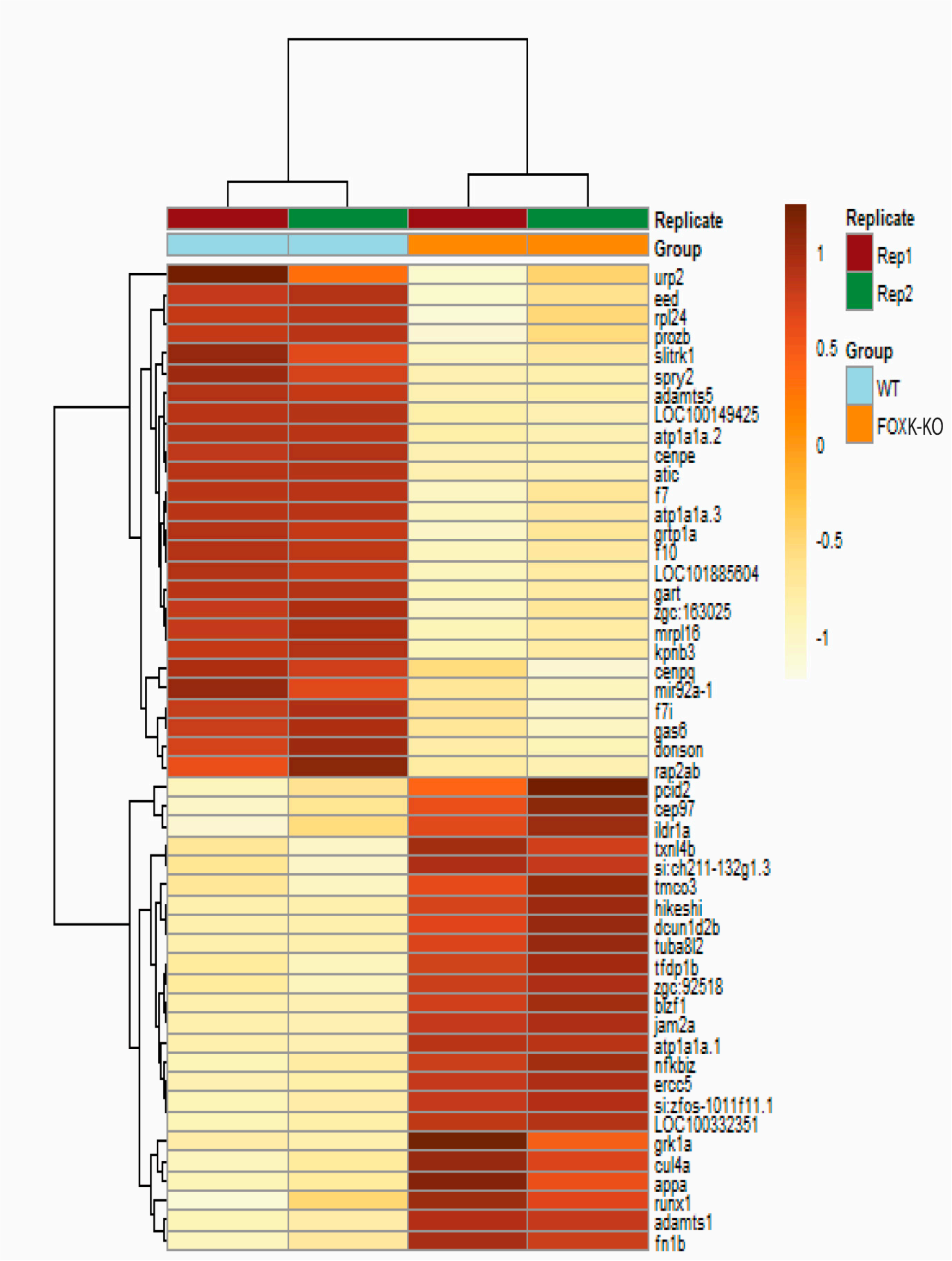

Supplementary Fig S1. Heat map analysis showing the top differentially expressed genes in WT vs Foxk KO zebrafish embryo. The Hierarchical clustering analysis shows the down-regulated and up-regulated genes among the wild type and Foxk knockout zebrafish embryo.

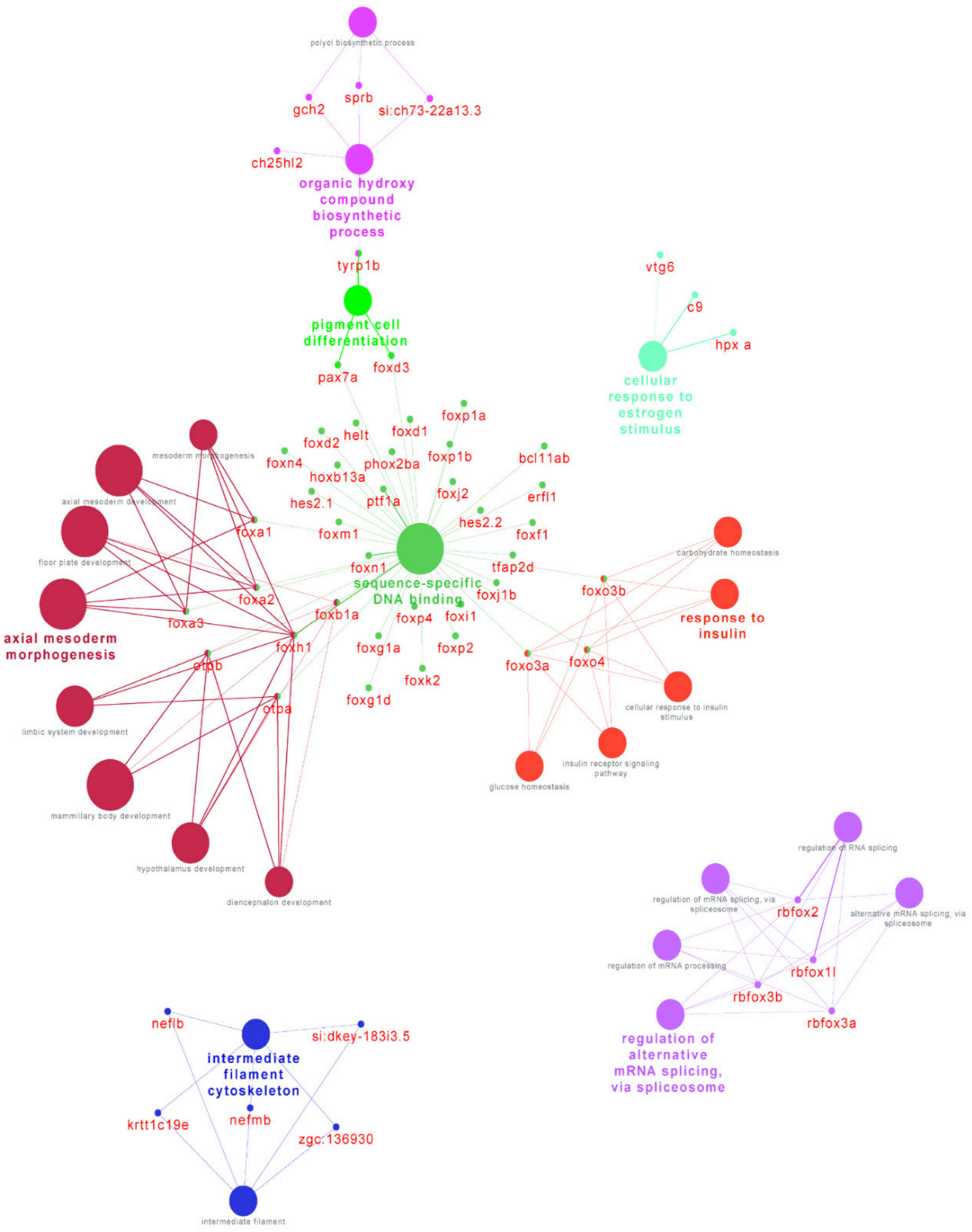

Supplementary Fig S2. ClueGo visualization of a gene ontology interaction network. The ClueGo plot analysis shows the down-regulated and up-regulated genes involved in the network and signalling pathways of zebra fish embryos development.

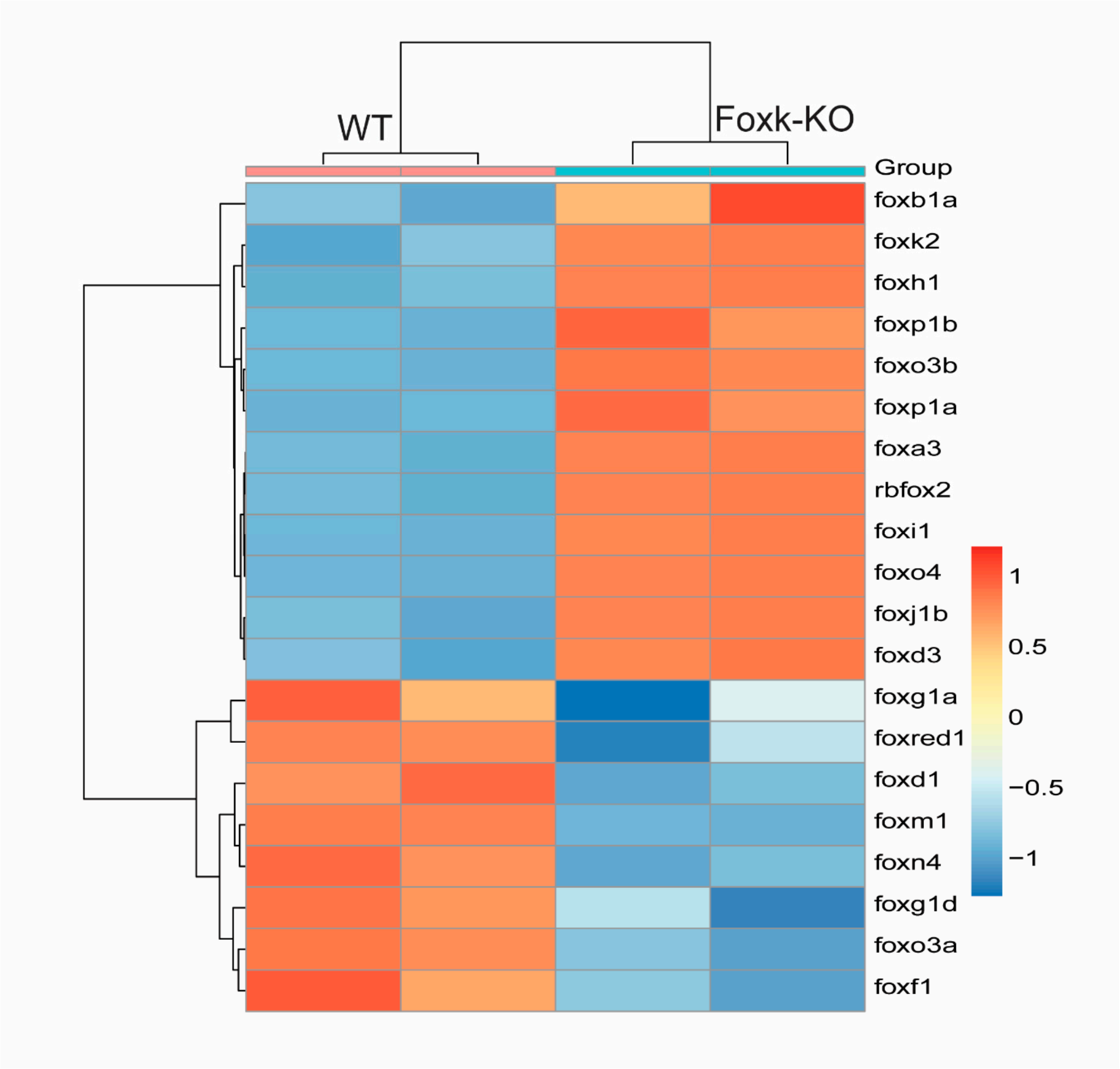

Supplementary Fig S3. Heat map analysis showing the top differentially expressed Foxo family associated genes in comparison to wild type and Foxk KO. The Heat map shows the down-regulated and up-regulated genes involved in Foxo family pathways of zebra fish embryos development.

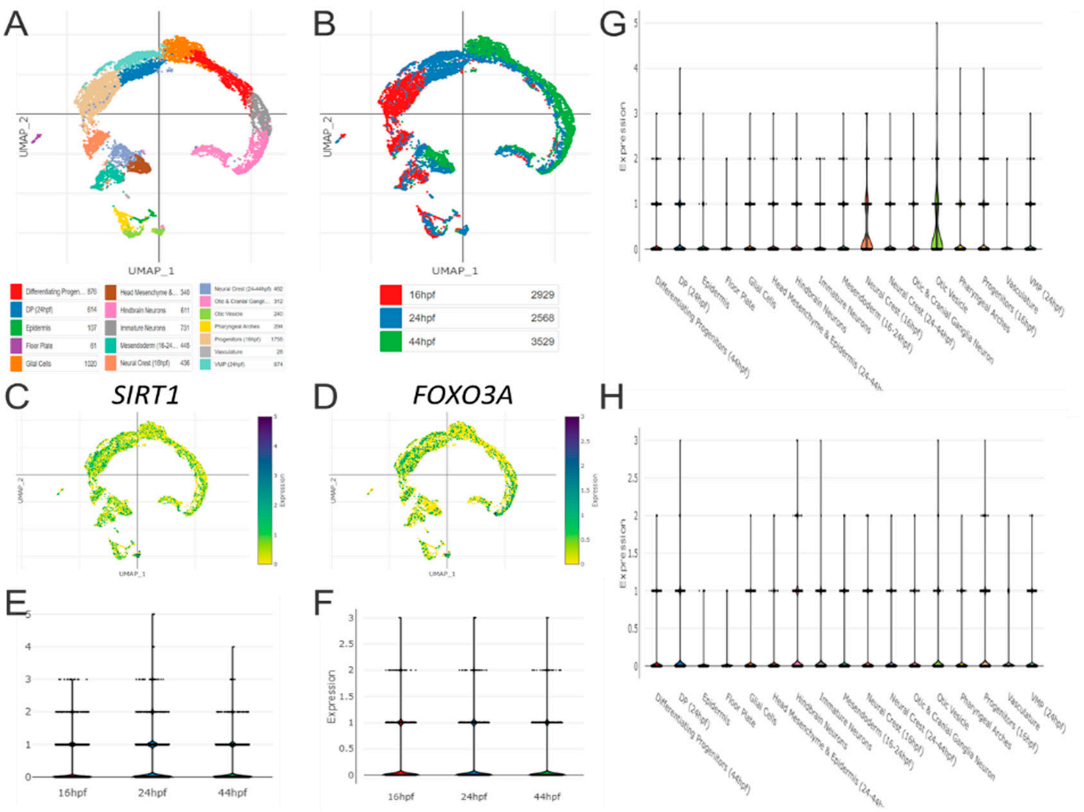

Supplementary Fig S4. Single cell transcriptomic analysis of the zebrafish hindbrain. (A, B) UMAP showing clusters of different cell types (A), and cell population involved in different developmental stages; 16, 24, and 44 hpf. **(C-F)** UMAPs (C, D) and violin plots (E, F) showing the expression of SIRT1 (C, E) and FOXO3A (D, F) in cell population involved in different developmental stages. **(G, H)** Violin plots showing the cell type-specific expression level of SIRT1 (G) and FOXO3A (H).

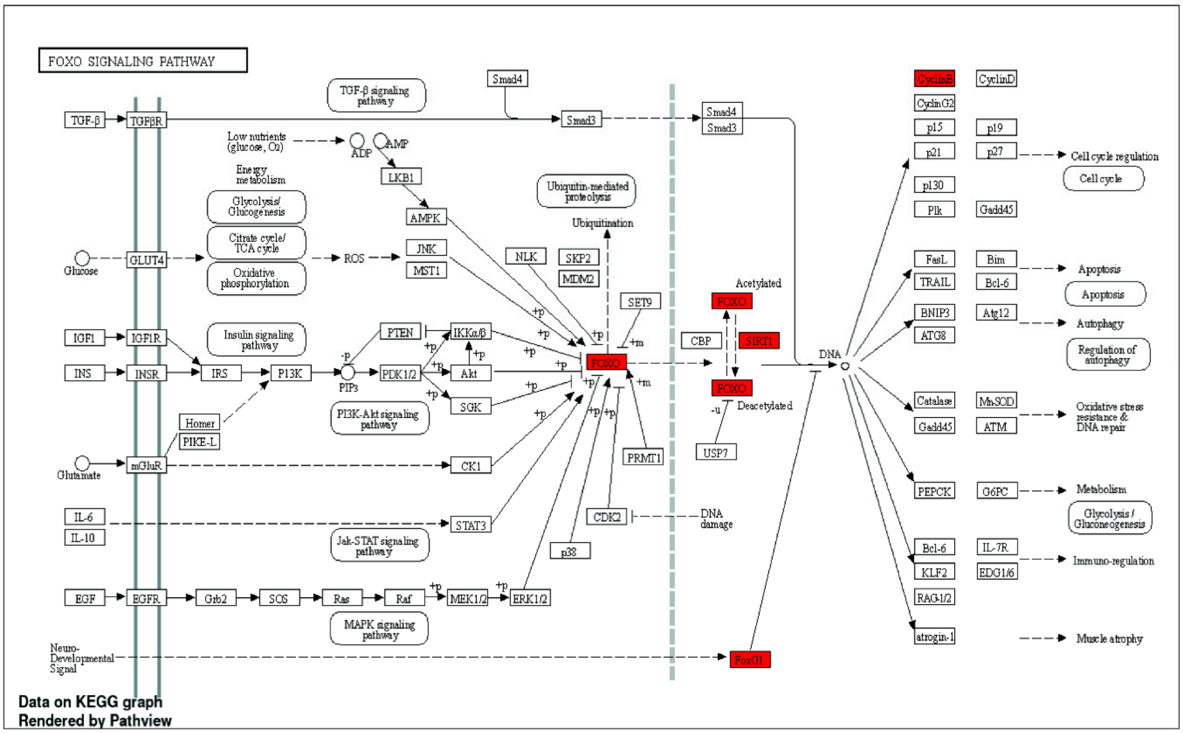

Supplementary Fig. S5. The KEGG pathway analysis in STRING for protein protein interaction illustrates the FOXO signalling pathway. The proteins involved in SIRT1/FOXO family for the development of zebra fish embryos is shown in the KEGG pathway map. This SIRT1/FOXO signaling pathway controls and regulates the signaling proteins related to cell-cycle control, apoptosis, oxidative stress resistance, glucose metabolism and life longevity. SIRT1 and FOXO interact to maintain the homeostasis and the cellular functions of oxidative stress and autophagy.
